# To Be or Not to Be a Germ Cell: The Extragonadal Germ Cell Tumor Paradigm

**DOI:** 10.3390/ijms22115982

**Published:** 2021-06-01

**Authors:** Massimo De Felici, Francesca Gioia Klinger, Federica Campolo, Carmela Rita Balistreri, Marco Barchi, Susanna Dolci

**Affiliations:** 1 Department of Biomedicine and Prevention, University of Rome Tor Vergata, 00133 Rome, Italy; klinger@uniroma2.it (F.G.K.); marco.barchi@uniroma2.it (M.B.); 2 Department of Experimental Medicine, University of Rome La Sapienza, 00161 Rome, Italy; federica.campolo@uniroma1.it; 3 Department of Biomedicine, Neuroscience and Advanced Diagnostics (Bi.N.D.), University of Palermo, 90133 Palermo, Italy; carmelarita.balistreri@unipa.it

**Keywords:** germ cell tumor, primordial germ cells, germline, EG cells

## Abstract

In the human embryo, the genetic program that orchestrates germ cell specification involves the activation of epigenetic and transcriptional mechanisms that make the germline a unique cell population continuously poised between germness and pluripotency. Germ cell tumors, neoplasias originating from fetal or neonatal germ cells, maintain such dichotomy and can adopt either pluripotent features (embryonal carcinomas) or germness features (seminomas) with a wide range of phenotypes in between these histotypes. Here, we review the basic concepts of cell specification, migration and gonadal colonization of human primordial germ cells (hPGCs) highlighting the analogies of transcriptional/epigenetic programs between these two cell types.

## 1. Introduction

In mammals, the unique core regulatory circuitry of transcriptional factors, epigenetics and signaling inputs regulating germline specification is becoming revealed. 

In the present work, we critically review old and new evidence that the unique pluripotency status is imposed on hPGCs by epigenetics traits regulated by pluripotency, and germline transcription factors that make these cells prone to undergo tumorigenesis. Such notion is discussed under the view that PGCs might be part of a pluripotent stem/progenitor cell pool exhibiting common markers that contribute to various somatic cell lineages (see also [1,2,3]).

## 2. Extragonadal Germ Cell Tumors

Germ cell tumors represent neoplasms derived from germ cells that can contain both immature and mature elements differentiating into several tissue types. Gonads are the preferred location for the onset of these tumors; however, they can be also found in extragonadal sites (EGCTs) accounting for 1–5% of all germ cell tumors. The most widely accepted theory suggests that these tumors arise from PGCs, the embryonic precursors of adult gametes, misplaced during their migration to gonads. While extragonadal localizations likely represent primary sites before puberty, EGCTs are considered metastases from occult or “burned out” gonadal cancers when they occur in young adults. 

PGCs migrate across the embryo to reach developing gonads, where they differentiate into gametes. These cells require an intrinsic motility program and external guidance cues to survive and successfully migrate. Proper guidance involves both attractive and repulsive cues, expression of a variety of adhesion molecules and is mediated by protein and lipid signaling [4]. In addition to the obvious effect of disrupted PGC migration on fertility, aberrant movement to ectopic sites is one mechanism that could account for the development of EGCTs in humans.

Gunnar Teilum first proposed a theory concerning the malignant potential of extragonadal germ cells known as the “germ cell theory”, suggesting that EGCTs originate from stray PGCs, which have undergone a malignant transformation during embryonic development [5]. Several studies on mammals revealed the presence of PGCs in extragonadal tissues long after gonadal differentiation, showing that extragonadal PGCs can last for some time. In some cases, PGCs observed in the adrenal glands [6,7], not only survive, but also in both genders differentiate and mature into oocytes [8].

EGCTs are usually seen in children or young adults and typically arise in midline locations. In adults, the most common sites of primary EGCTs are, in descending order, the mediastinum, retroperitoneum, and cranium [9], while in children, the cranium and sacrococcygeal region are the common sites [9,10,11,12].

Genetic conditions such as Klinefelter syndrome (KS), Marfan syndrome or Down syndrome, as well as somatic mutations of genes involved in PGC proliferation have been shown to associate with EGCTs, which can appear either in childhood or in the post-puberal age [13,14,15,16]. 

Histologically, EGCTs comprise seminomas (SEs), also known as germinoma, and nonseminomatous tumors (NSTs). The latter includes teratoma, embryonal carcinoma, endodermal sinus tumor (yolk sac tumor), choriocarcinoma, and tumors with mixed histology. SEs are composed of a homogeneous population of neoplastic gonocytes. NSTs are more aggressive with heterogeneous histological features, including partially differentiated populations [17]. Nonseminomas may contain a pluripotent component known as embryonal carcinoma (EC). EC cells are considered to be a malignant variant of embryonic stem cells (ES) because they share many morphological and biochemical features of cells derived from the inner cell mass [18]. Most of the germ cell tumors with the exception of teratoma and yolk sac tumors are believed to originate from precursor cells termed carcinoma in situ (CIS) or more specifically germ cell neoplasia in situ (GCNIS), representing transformed PGCs or gonocytes [19].

## 3. Germline Specification and PGC Migration

The genome of the germ cells, which is the only pathway to the next generation, must be maintained in an epigenetically reprogrammable status that allows at the same time the formation of unipotent gametes. In the mammalian embryo, the specification of the germline is inductive and requires a complex core regulatory circuitry of transcription factors locally activated by a specific combination of growth factors. During the last ten years, the genetically specified program of transcriptional activity governing the germline specification and determination in mammals has been intensely studied. Much of what is known today about mammalian germline development comes from work performed on mouse models. Recent studies revealed, however, critical species-specific differences between mouse and human PGC development, including differences in the transcription factor network [20,21]. 

### 3.1. Specification

**Figure 1:**
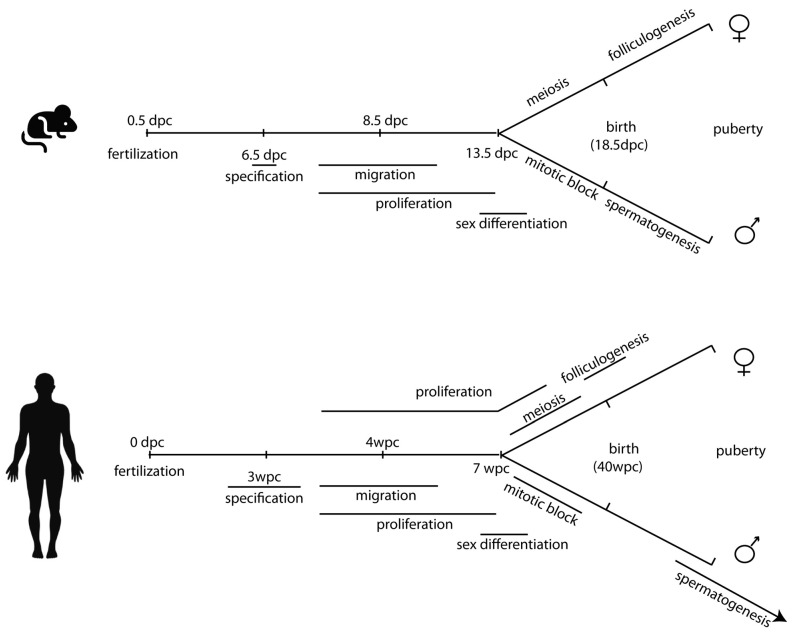
Parallel timetable of germ cell development in mice and humans.

Germ cell specification occurs between wpc (week post conception) 2 and 3 in humans, at 6.5 days post coitum/conception (dpc) in mice (see Figure 1 for a parallel timetable of mice and human germ cell development). To circumvent the technical and ethical barriers that limit the understanding of the mechanisms that regulate such process in humans, surrogate cell culture models have been recently generated from human pluripotent stem cells. As a matter of fact, pluripotent stem cell-derived human primordial germ cell-like cells (hPGCLCs) in vitro provide important opportunities to study this process. 

These studies revealed that hPGCs likely originate from a lineage-primed progenitor expressing the transcription factor TFAP2A. At around day post conception (dpc) 11, in the implanted blastocyst, under the action of the growth factor BMP4, TFAP2A^+^ cells are specified from epiblast cells possessing a transitional pluripotent state, called “germinal pluripotency”, that shares characteristics with pre- (ground state naive pluripotency) and post-implantation (primed state) epiblasts [22]. TFAP2A^+^ progenitors transiently expressing the mesodermal/endodermal transcription factors GATA3, EOMES, and T (also known as BRACHYURY), have the potential for germ cell specification but also for differentiation into multiple somatic fates including amnion and cells from the gastrulating embryo. In this contest, prolonged exposure to BMP4 induces another transcription factor, TFAP2C that operates in the TFAP2A^+^ progenitors, upstream of PRDM1 (BLIMP1) to regulate the expression of the germ cell fate determinant SOX17, while simultaneously preventing germline cells to become somatic cells and re-wiring pluripotency in the newly specified PGCLCs to a naive-like state. A specific role of TFAP2C in humans involves the opening of naive-specific enhancers and the acquisition of naive-like pluripotency [23]. While in vitro LIF, KL and EGF, are necessary to specify PGCLCs, it is unknown whether other factors in vivo are required to fully specify SOX17/NANOS3 expressing PGCs. In this regard, PRDM14 critically cooperates with TFAP2C and PRDM1 to upregulate/maintain germ cell (SOX17/NANOS3) and pluripotency (OCT4/NANOG/KLF4) genes, while repressing WNT signaling and somatic markers. In this contest, PRDM1 seems crucial to suppress SOX17-induced expression of endodermal genes as well as BMP- or WNT-induced expression of mesodermal genes [21,24]. 

Seminomas typically express SOX17 in their nuclei and are negative for SOX2, known to be not expressed in human PGCs [25], while EC cells express SOX2 and are negative for SOX17 [26]. Both types of tumors express OCT4 and NANOG [27,28], while positive staining for TFAP2C, NANOS3 and OCT4 through immunohistochemical (IHC) is used for the recognition of GCNIS [29,30]. *Lin28a* promotes mouse PGC development in vivo and in vitro via the *let-7* precursor microRNA that targets PRDM1 [31]. While *Lin28a* knockdown compromises the size of the germ cell pool both in males and females [32], its overexpression associates with human germ cell tumors [33]. 

### 3.2. Migration

After specification, PGCs migrate along the hindgut and through the dorsal mesentery to the gonadal anlage [4]. 

In the human embryo, the dorsal part of the yolk sac containing the PGCs, becomes incorporated in the developing gut during the lateral folding 4 wpc, indicating that PGC translocation into the gut may be passive [34]. Either way, questions are left open concerning how PGCs are displaced at this early stage. In the human embryo at around 35 dpc it was recently shown that PGCs are present along autonomic nerve fibers and Schwann cells from the dorsal mesentery to the gonad, where they seem to be delivered via fine nerves [35,36]. The migration is accompanied by a wave of stem cell factor/kit ligand (SCF/KITL) expression by the surrounding somatic cells, that is suggested to promote migration, survival and proliferation of PGCs [37]. In the presence of KITL, PGCs close to the gonadal anlage continue to migrate and colonize the gonads, while PGCs remaining further away in the midline body structures normally die by the *Bax*-dependent apoptotic pathway in the absence of KITL [37,38,39,40]. At the same time NANOS3 expression sustains the germ cell lineage by suppressing both Bax-dependent and Bax-independent apoptotic pathways [41]. 

During this transit, continuous epigenetic remodeling leads to a progressively global erasure of genomic imprints. Actually, gonadal hPGCs show the lowest levels of DNA methylation at CpG islands observed in the human genome to date [42]. This demethylation also involves transposons with the exception of a significant fraction of LINE-1 retrotransposable elements (L1-TEs). Su and colleagues found that L1 protein 1 (also defined as L1-p40) was expressed in all cases of childhood GCT examined [43]. Heterogeneity at a single cell level, however, suggests that only a fraction of human fetal germ cells express L1-TEs and utilize the PIWI-piRNA pathway to repress transposable elements by H3K9me3-mediated silencing, whereas other germ cells remain resistant [44]. The chromatin of migratory and gonadal hPGCs is characterized by low H3K9me2 and high H3K27me3 and H3K9me3 marks suggesting that these latter may be the key factors repressing constitutive heterochromatin in such cells. Moreover, in contrast to the mouse, hypomethylation of imprints in humans seems to occur before PGCs colonize the gonads and is maintained until at least 19 weeks of development. Similarly, while in mouse PGCs the inactive X chromosome is reactivated between E8.5 and E12.5, X reactivation in hPGCs occurs prior to 4 weeks of development. 

Interestingly, Mallol and colleagues [45] recently found that global upregulation of the repressive H3K27me3 mark is PRDM14 dosage-dependent in mouse PGCs of both sexes and that at the same time it is also required for removal of H3K27me3 from the inactive X-chromosome. This evidence reinforces the notion that transcription factor and epigenetic programs are strictly related. 

Both mPGCs and hPGCs proliferate during migration and after reaching genital ridges (GRs), where they become surrounded by cords of somatic cells. The regulation of hPGC proliferation in both sexes in vivo is poorly understood. Data obtained from in vitro studies of human PGCs cultured on cell monolayers indicated that hPGCs are responsive to the same compounds (forskolin, retinoic acid) and growth factors (KITL, basic fibroblast growth factor, bFGF, or leukemia inhibitor factor, LIF) known to control survival and/or proliferation of mPGCs [46]. Most importantly, like mouse PGCs, human PGCs give rise to pluripotent embryonic germ cells (EG cells, when cultured in vitro in the presence of a cocktail of compounds and growth factors, see next section), suggesting that the mechanisms controlling PGC growth in mammals are largely conserved. 

Next paragraph focuses on the latent pluripotency derived from specification and how the highly hypomethylated status of chromatin imposed on PGCs during migration represents a risk for tumorigenic transformation.

## 4. The EG Cell Case

As for other embryonic cells, growth factors are critical determinants for the program of transcriptional activity and of the epigenetic changes occurring in germline specification and during PGC migration. If PGCs fail to reach their destination, the combination of the same growth factors in different microenvironments could allow their survival and induce their transformation.

It is likely that on way to the gonad anlage, PGC fate depends on whether they can preserve their identity by repressing differentiation cues towards somatic lineages. Multiple lines of evidence suggest that PGC fate remains undetermined until they colonize the gonads where they progressively stop expressing pluripotency markers, lose their somatic potential, and acquire the germ cell fate, undergoing meiosis to produce oocytes in females or mitotic arrest to give rise to prospermatogonia in males. DAZL, DDX4, MAEL, and TDRD12 have been reported to be transcription factors crucial to determine the germ cell fate within the gonads (see, for example, [47,48]).

Such latent pluripotency has been elegantly demonstrated by in vitro transformation of mouse or human PGCs in the presence of a cocktail of growth factors, such as Kitl, Lif, and bFgf [46,49,50], to form pluripotent stem cells, known as EG cells, that are capable to form teratomas when transplanted in the animal.

One of the first mechanisms responsible for mouse germ cell transformation is the deregulation of cell cycle/survival signals linked to overactive Pi3k/Pten/Akt pathway [51,52,53] followed by up regulation of c-myc, Klf4, and Stat3 [54,55]. Moreover, epigenetic changes controlled by Prdm1, Prdm14, and histone acetylation were found to be prerequisites for such transformation [54]. The use of chemical inhibitors of GSK3β or Tgfβ receptor and Erks or the Sox2 replacement SB431542 and the Klf4 replacement Kempaullone (generally called 2iL or R2iL) in combination with Lif preceded by short induction with bFgf and Kitl, could substitute the original protocol and even improved the efficiency of EG cell derivation [56]. Transient transfection with only one of the Yamanaka’s factors either Oct4, or Sox2, or Klf4 or c-myc in combination with Lif was also capable to transform PGCs into EG cells [57]. Intriguing, these latter transformation protocols resemble those used for reprogramming primed mouse epiblast stem cells (EpiSCs) and human ESCs into naïve ground pluripotency (for a review, see [58]).

Another molecule regulating the conversion of mouse PGCs to pluripotent cells is the RNA-binding protein, Dnd1. Deficiency in the *Dnd1* gene in the 129Sv genetic background causes testicular teratoma at high rates [59]. Dnd1 inhibits the interaction of microRNAs with their target messenger RNAs [60]. Several studies demonstrated that a number of molecules are involved in teratoma formation caused by deficiency in the *Dnd1* gene; moreover, enhancement of the cell cycle and suppression of apoptosis are likely key events for the conversion of PGCs into teratoma. For example, *p21cip1* and *p27kip1* mRNAs, which encode negative regulators of the cell cycle, were identified as targets of Dnd1, and a deficiency of Dnd1 resulted in downregulation of the p21cip1 and p27Kip proteins [61]. Interestingly, Dnd1-mediated epigenetic control of teratoma formation has been recently reported [62]. Mutation in *Dnd1* resulted in downregulation of Ezh2, a core member of polycomb protein complex 2 (Prc2), responsible for catalyzing H3K27 tri-methylation in PGCs forming teratoma. As a consequence, Cyclin D1 was upregulated and the PGC cell cycle dysregulated. In fact, it is known that this cyclin forms a complex with CDK4/6 and enhances the G1-S transition of the cell cycle in response to mitotic signals. Similar changes in the expression of these molecules were found in PGCs transformed in vitro in EG cells [61]. Although mutations in *DND1* in humans are unlikely to contribute significantly to germ cell tumors [63], these results support the notion that epigenetics alterations can be one of the causes of PGC transformation into tumorigenic cells.

## 5. TFAP2C and PRDM14

In the epigenetic reprogramming of the human germline a central role is played by the transcription factors TFAP2C and PRDM1. 

The TFAP2C protein can act as either a homodimer or heterodimer with other family members and plays important roles in the regulation of both embryonic development and carcinogenesis including germinoma. In the mouse, loss of Tfap2c leads to derepression of somatic programs, and loss of germ cells. In addition, in such condition, the expression of genes involved in germ cell maintenance and differentiation such as *Stella*, *Dazl*, *c-Kit*, *Cxcr4* and *Nanos3*, are severely impaired [64]. Moreover, Tfap2c is induced during the generation of iPSCs from mouse fibroblasts and acts as a facilitator for iPSCs formation [65]. E-cadherin is an important downstream target of TFAP2C [65] and in the mouse embryo E-cadherin-mediated cell–cell interaction among cells containing PGC precursors is essential for directing such cells to the germ cell fate [66]. Of note, the formation of cell aggregates is a prerequisite for induction of PGCLCs from pluripotent stem cells both in mouse and humans [67]. In humans, as reported above, TFAP2C operates in the TFAP2A^+^ PGC progenitors, upstream of PRDM1, to upregulate the expression of the germ cell fate determinant SOX17, while simultaneously preventing germline cells to become somatic cells and reprogramming pluripotency to a naive-like state. Although the factor/s that induce TFAP2C in the human embryo are not known, its induction in vitro is mainly driven by BMP4. In line with this role in hPGC specification, TFAP2C is upregulated during primed to naïve reversion of hESCs and becomes widely distributed along naïve-specific enhancers [68]. It is likely that upon expression, TFAP2C, as other AP-2 family members, requires an unmethylated, open, and accessible chromatin structure for optimal DNA binding and transactivation [69]. 

Relevant to the subject covered by the present paper, high levels of TFAP2C protein were observed in precursor lesions of CIS and classical seminomas [70,71].

PRDM14 is a member of the PR domain-containing family. There are 16 PRDM proteins, many of which are known as master epigenetic regulators of cellular differentiation, especially in hematopoietic, epithelial, and neural cells. PRDM proteins are among the most widely deregulated proteins involved in the early transformation of normal cells to cancer cells and their expression has been shown to be related to cancer properties such as proliferation, drug resistance, and differentiation.

PRDM14 is characteristically expressed in pluripotent stem cells where it exhibits multiple functions, including a scaffold for chromatin remodeling, a transcription regulator required for maintaining pluripotency, and a required component for epigenetic reprogramming. In mouse ES cells, Prdm14 ensures naive pluripotency through a dual mechanism: antagonizing activation of the FgfR signaling by the core pluripotency transcriptional circuitry, and repressing expression of de novo DNA methyltransferases that modify the epigenome to a primed epiblast-like state. Prdm14 exerts these effects by recruiting polycomb repressive complex 2 (Prc2) specifically to key targets and repressing their expression. Prdm14 over-expression increases activation rates of the Pi3k/Akt/mTorc1 cell proliferation pathway, blockage of apoptotic pathways and down-regulation of the cyclin inhibitor p21. Prdm14 is essential for mouse PGC specification by assisting Blimp1-mediated repression of somatic transcripts, initiating global epigenetic reprogramming and upregulating germline-specific genes, in particular Nanos3. Accordingly, Prdm14 is sufficient to induce the PGC state in EpiLCs and its deletion causes proliferation and differentiation defects in PGCLCs [72]. 

As reported above, Mallol and colleagues [45] recently found that global upregulation of the repressive H3K27me3 mark is Prdm14 dosage-dependent in mouse PGCs of both sexes and that this transcription factor is also required for removal of H3K27me3 from the inactive X-chromosome supporting the notion that transcription factors and epigenetics are strictly related.

**Figure 2:**
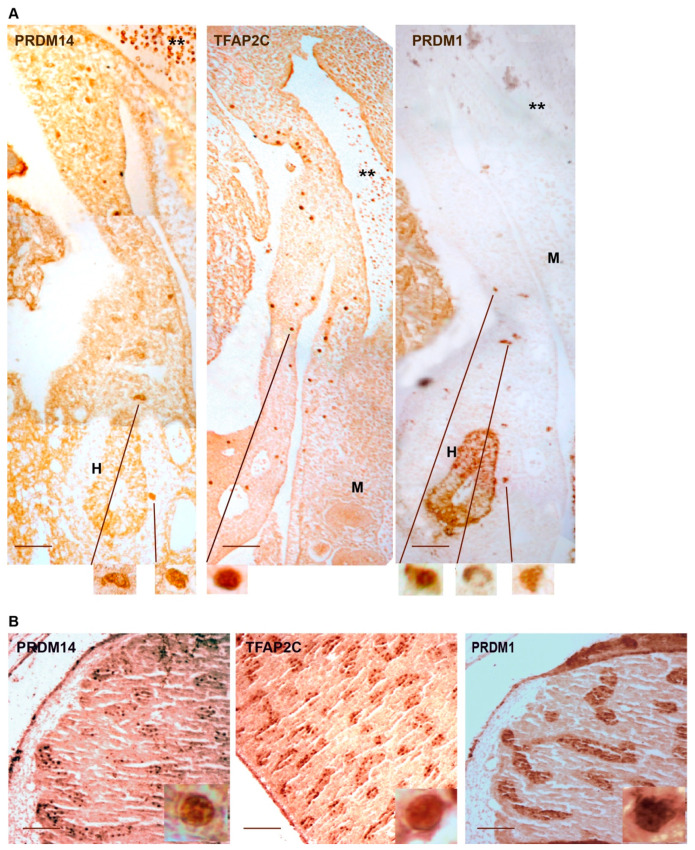
Expression of PRDM14, TFAP2C, and PRDM1 in human germ cells. (**A**) Section of a human embryo at 4 wpc carried at the level of dorsal mesentery and stained for TFAP2C, PRDM14, and PRDM1. Lines point to magnified PGCs captured during migration along the dorsal mesentery that show nuclear PRDM14 or TFAP2C staining while PRDM1 is found in multiple localizations. **—dorsal aorta; H—hindgut; M—mesonephros. (**B**) Fetal testis at 11 wpc stained for PRDM14, TFAP2C, and PRDM1. Insets represent nuclear localization signals in gonocytes. Bars represent 100 μm. Inset bars represent 10 μm. Two human fetuses, obtained under informed consent from pregnant women undergoing therapeutic abortion at 4 and 11 wpc, respectively, at the Dipartimento di Promozione della Salute, Materno-Infantile, Medicina Interna e Specialistica di Eccellenza “G. D’Alessandro” (PROMISE) of the University of Palermo. The ethical committee of the Department of Biomedicine, Neuroscience and Advanced Diagnostics of the University of Palermo, Italy approved the use of human fetal tissue for research purpose. Mouse anti-TFAP2C (sc-12762) was from SantaCruz Biotecnology; Mouse anti-PRDM1 (MA1-16874) and rabbit anti-PRDM14 (PA5-93019) were from Thermofisher. Dako EnVision dual link system-HRP (Agilent) was used to reveal primary antibodies.

Although it has been shown to play an important role in hPGCLC specification [73], PRDM14 expression in vivo has been only documented in gonadal hPGCs, where it shows both cytoplasmic and nuclear localization [20,73]. We found that also migratory hPGCs expressed PRDM14, by probing sections from an ectopically implanted early human embryo at 4 wpc with PRDM14 antibodies. PRDM14 was expressed in the nuclear compartment of hPGCs scattered along the dorsal mesentery similarly to TFAP2C. On the contrary, we found that PRDM1 was localized in different combinations, such as nuclear, cytoplasmic or both (Figure 2A). Interestingly, male gonadal germ cells at 10 weeks of gestation still showed nuclear localization for the three transcription factors (Figure 2B, [20,74]). PRDM14 is highly expressed in embryonal carcinoma cell lines, embryonal carcinomas, seminomas, intracranial germinomas, and yolk sac tumors, but is not expressed in teratomas and its overexpression causes proliferation and differentiation defects in hPGCLCs [75].

## 6. Remarks and Conclusions

**Figure 3:**
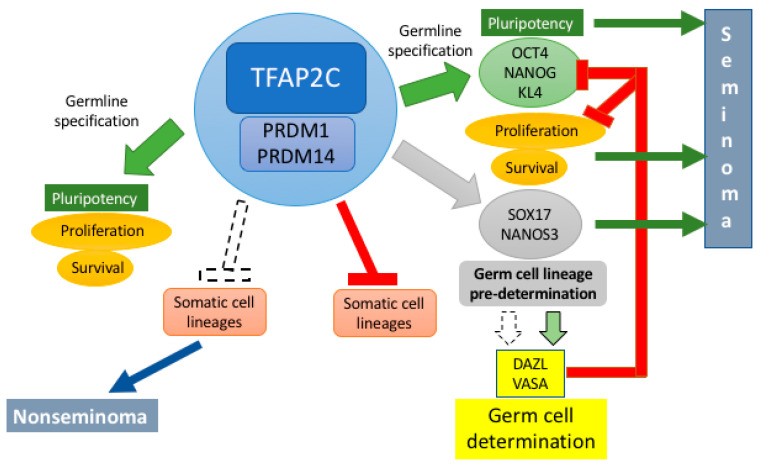
Schematic and simplistic hypothesis of the transcription factor core circuits crucially involved in the specification and determination of the human germline and of the possible origin of the extragonadal germ cell tumors discussed in the present review. Briefly, the expression of transcription factors TFAP2C, PRDM1, and PRDM14, necessary for the germline specification, control the pluripotency transcription factors (OCT4, NANOG, KL4) and associated proliferation/survival circuits. At the same time TFAP2C, PRDM1, and PRDM14 inhibit somatic cell lineages; subsequently, SOX17 and NANOS3 direct the PGC precursors to a pre-determined germ cell stage. Within the gonads, DAZL and VASA complete PGC determination as germ cells and repress pluripotency and proliferation while TFAP2C, PRDM1, and PRDM14 are progressively downregulated. Seminoma could arise (green arrows) when PGC determination is not completed (dotted arrow), the survival maintained and proliferation prolonged or, under certain condition, re-established (green arrows); conversely, nonseminoma could arise (blue arrow) if PGCs remain only specified, the inhibitory action of TFAP2C, PRDM1, and PRDM14 on somatic cell lineage differentiation not maintained, the survival maintained and proliferation prolonged or, under certain condition, re-established.

On the basis of the evidence reported here, we postulate that up-or down-regulation of transcription factors, in particular of TFAP2C and PRDM1, in misplaced PGCs, resulting from or causing altered epigenetics germline program, might be among the major causes for GCT formation. It is surely not a coincidence that PGCs can be reprogrammed to pluripotency in culture without genetic manipulation. The variety of characteristics of these tumors support the notion that they could basically derive from germ cells that remain incompletely determined into the germline (seminoma) with a dysregulated cell cycle or from germ cells in which somatic differentiation programs are not maintained in the repressed state (nonseminomas) (Figure 3).

Alternatively, we speculate that in microenvironment where PGCs do not receive any differentiation signals but remain viable, as their own fate determinants are gradually depleting, the cells cannot assume a specific fate. Under these conditions the PGCs possessing a latent pluripotent state are susceptible to transformation. Although they are mainly eliminated by cell death for the prevailing expression of cell death factors (probably BAX) over cell survival factors (perhaps BCL-X) [76], in some microenvironments they can survive for a long period of time and without differentiating. For example, if they remain in the presence of the known cell survival signaling mediated by KITL/KIT interaction [36,77].

The progression and eventual detection of the tumors occurs decades later in humans. Long-term maintenance of pluripotent stem cells in a naïve state may increase risk of chromosomal anomalies [78]. One of the most consistent features of the germ cell tumors, in particular of testicular germ cell tumors (TGCTs), is the gain of material in the short arm of chromosome 12, that occurs in almost 100% of TGCT cases; 80% of them involve the formation of an isochromosome of the short arm (12p). The mechanism that associates the gain of 12p to the development of invasiveness is not yet well understood but it is believed that dysregulation of a number of genes therein contained are involved, including DPPA3/STELLA, SOX5, PHC2, ATF7IP, and proto-oncogenes Cyclin D2 and KRAS [79].

Reflecting this “ambivalence” of the core regulatory circuitry, early PGCs could fluctuate from a naive and primed state regulated by transcriptional circuitry and signaling inputs. 

Associated to these remarks is the question of why such apparently risky strategy for PGC specification and determination has been selected during mammalian evolution. Many invertebrates and nonmammalian vertebrates express cytoplasmic germ plasm determinants at or soon after PGC specification instead of after gonad colonization. Is it possible that the extended duration of mammalian PGC migration benefits from an uncommitted pluripotent status, perhaps to counteract somatic development? Does the pluripotent state of PGCs facilitate cell migration? Or do mammalian PGCs postpone germ cell determination to contribute to non-gametogenic lineages? 

Of note, PGCs are suggested to have a pluripotent stem cell potential that may give rise to hematopoietic stem cells, that originate in the aorta-gonad-mesonephric region [80,81] and perhaps to muscle cells [82]. Actually, PGCs have also been found in high numbers in the para-aortic tissue, liver, and branchial arches in the pig [6]. In humans, primary sites of ectopic germ cells are within the CNS, but they are also seen in the pelvic region, the mediastinum, and thorax [83]. Intriguingly, PGCLCs were identified as primordial germ cell-like cells as liver metastasis initiating cells in mouse tumors models [84]; germ cell tumors and associated hematologic malignancies were reported to evolve from a common shared precursor [85], while PGCs have been proposed at the origin of mucinous cystic neoplasms of the pancreas and mucinous ovarian tumors [86]. In addition, NANOS3 expression normally restricted to germ cells has been detected in various human cancers [87] and reported to promote proliferation and migration of human glioblastoma cells [88].

All these results support the very old Simkins’ hypothesis [89], recently re-proposed by Mikedis and Downs [90] (2014), that the so called migratory PGCs are part of a pluripotent stem/progenitor cell pool that exhibits common markers and that, as actually occur in various species (see [47] and references herein), contribute to various somatic cell lineages and/or even to the stem cell population of adult tissues. Further studies are certainly welcome to clarify these intriguing aspects of the mammalian development.
